# Prognostic microRNA signatures derived from The Cancer Genome Atlas for head and neck squamous cell carcinomas

**DOI:** 10.1002/cam4.718

**Published:** 2016-04-25

**Authors:** Nathan Wong, Shariq S. Khwaja, Callie M. Baker, Hiram A. Gay, Wade L. Thorstad, Mackenzie D. Daly, James S. Lewis, Xiaowei Wang

**Affiliations:** ^1^ Department of Radiation OncologyWashington University School of MedicineSt. LouisMissouri; ^2^ Department of Biomedical EngineeringWashington UniversitySt. LouisMissouri; ^3^ Department of Pathology and ImmunologyWashington University School of MedicineSt. LouisMissouri; ^4^ Department of Pathology, Microbiology and ImmunologyVanderbilt University Medical CenterNashvilleTennessee

**Keywords:** Gene signature, head and neck cancer, microRNA, oropharyngeal cancer, prognosis, The Cancer Genome Atlas

## Abstract

Identification of novel prognostic biomarkers typically requires a large dataset which provides sufficient statistical power for discovery research. To this end, we took advantage of the high‐throughput data from The Cancer Genome Atlas (TCGA) to identify a set of prognostic biomarkers in head and neck squamous cell carcinomas (HNSCC) including oropharyngeal squamous cell carcinoma (OPSCC) and other subtypes. In this study, we analyzed miRNA‐seq data obtained from TCGA patients to identify prognostic biomarkers for OPSCC. The identified miRNAs were further tested with an independent cohort. miRNA‐seq data from TCGA was also analyzed to identify prognostic miRNAs in oral cavity squamous cell carcinoma (OSCC) and laryngeal squamous cell carcinoma (LSCC). Our study identified that miR‐193b‐3p and miR‐455‐5p were positively associated with survival, and miR‐92a‐3p and miR‐497‐5p were negatively associated with survival in OPSCC. A combined expression signature of these four miRNAs was prognostic of overall survival in OPSCC, and more importantly, this signature was validated in an independent OPSCC cohort. Furthermore, we identified four miRNAs each in OSCC and LSCC that were prognostic of survival, and combined signatures were specific for subtypes of HNSCC. A robust 4‐miRNA prognostic signature in OPSCC, as well as prognostic signatures in other subtypes of HNSCC, was developed using sequencing data from TCGA as the primary source. This demonstrates the power of using TCGA as a potential resource to develop prognostic tools for improving individualized patient care.

## Introduction

Head and neck squamous cell carcinoma (HNSCC) constitutes approximately 3% of all cancer diagnoses in the United States, with about 45,000 new cases in 2015 1. Among head and neck cancers, oral cavity, oropharyngeal, and laryngeal cancers are the most common, accounting for 24%, 23%, and 27% of all diagnosed cases, respectively 2.

Due to the heterogeneity of these subtypes of HNSCC, a single prognostic signature identifying high‐ and low‐risk patients cannot be generated to cover all types of HNSCC. However, multiple studies have indicated that individual biomarkers can stratify high‐risk and low‐risk patients within the various subtypes 3, 4, 5. These biomarkers are not limited to coding genes. Included among the proposed biomarkers are microRNAs (miRNAs), which are short single‐stranded RNA sequences (~22 n.t.) that function in post‐transcriptional regulation. Further studies have shown that within oropharyngeal cancer, infection by human papillomavirus (HPV) is a favorable prognostic marker 6. Greater prognostic power has been attained by combining groups of biomarkers into a single signature for different subtypes of HNSCC, with various degrees of success 7, 8.

The identification of novel biomarkers and subsequent development of prognostic signatures requires in‐depth analysis of genetic profiles. For example, high‐throughput gene expression profiling data have been made available by The Cancer Genome Atlas (TCGA), a joint effort of the National Cancer Institute and the National Human Genome Research Institute to provide a comprehensive set of patient genetic profiles across multiple cancer types 9. This has extended to HNSCC, with a total of 529 HNSCC samples being made available 10. Included in the available data are RNA‐seq and miRNA‐seq profiles for the majority of the provided patient samples.

In this study, we investigated the prognostic value of miRNA biomarkers for oropharyngeal squamous cell carcinoma (OPSCC), oral squamous cell carcinoma (OSCC), and laryngeal squamous cell carcinoma (LSCC), using profiling data obtained from TCGA. These biomarkers were then used to develop unique prognostic signatures that robustly predicted overall survival in the respective subsets of HNSCC. We further demonstrate that use of the TCGA public dataset can provide a more general picture of head and neck cancer as the prediction models obtained can be applied to an independently obtained dataset. Through a combined analysis of TCGA data and independently generated data, we have provided an additional set of biomarker tools for the clinical setting that can assist in determining the best course of treatment for patients with head and neck cancer.

## Materials and Methods

### Retrieval of public data

A total of 523 anonymized patients in the TCGA database were identified as having primary HNSCC. The clinical patient files were downloaded from TCGA Data Portal (https://tcga-data.nci.nih.gov/tcga/). Of the 523 HNSCC patients, 82 patients had a primary tumor in the oropharynx, 313 patients had a primary tumor of the oral cavity, and 115 patients had primary tumors in the larynx. A cutoff of 5 years was applied to all patient survival data.

All gene sequences were downloaded from the UCSC Genome Browser 11. Index files mapping transcript accessions to NCBI Gene IDs were downloaded from the NCBI ftp site 12. All mature miRNA sequences were downloaded from miRBase 13. Raw miRNA‐seq data was obtained for 81 of the 82 OPSCC patients, 311 of 313 OSCC patients, and all of the laryngeal cancer patients. Raw RNA‐seq data was obtained for 72 of the 82 oropharyngeal cancer patients. All raw RNA‐seq and miRNA‐seq files were downloaded through the Cancer Genomics Hub 14.

### TCGA sequence analysis

Sequence alignment was performed using the Bowtie program 15. Raw miRNA‐seq reads were aligned to the human miRNome. The read counts were then normalized to reads per million reads mapped per sample and set to a floor value of 1 for lowly expressed miRNAs. Raw RNA‐seq reads were aligned sequentially to human RefSeq annotated sequences, the human reference genome, and the virome. The read counts were normalized to reads per kilobase per million mapped reads, and then to the 2000th gene before being set to a floor of five normalized reads for lowly expressed mRNAs. Both miRNA‐seq and RNA‐seq reads were subsequently log_2_ normalized.

### Statistical analysis for survival and correlation

Overall survival analysis was conducted using the “survival” package in R (http://www.r-project.org). Correlation and covariance analysis was conducted in MATLAB 16. Univariate Cox proportional hazards regression analyses were performed to evaluate the correlation between individual miRNAs or mRNAs with overall survival. The *P*‐values for outcome correlation were calculated using the Wald test. The final prognostic signatures were also evaluated in this manner. Multivariate Cox proportional hazards analyses were conducted to evaluate the independent prognostic value of the miRNA signature after controlling for common clinical variables. The Kaplan–Meier estimator was used to determine the empirical survival probabilities and *P*‐values from the log‐rank test indicated the significance of the miRNA prediction outcome model.

### Collection of independent validation data sets

A total of 95 OPSCC cases were included in this study for validation. Patients were treated at Washington University School of Medicine with definitive chemoradiation, or with primary surgery followed by radiation therapy with or without chemotherapy. Clinical data were collected from the patients and then updated retrospectively after follow‐up review.

For all 95 of the patients, formalin‐fixed, paraffin‐embedded (FFPE) tumor tissues were collected for pathological analysis before radiotherapy or chemotherapy. Sections from each case were stained with hematoxylin and eosin, and reviewed by a study pathologist at the Washington University to confirm the diagnoses. Tumor regions from each section were identified and macrodissection was conducted. Total RNA was extracted from the identified tumor regions using the miRNeasy FFPE kit (Qiagen, Hilden, Germany) according to the manufacturer's protocol. In total, 66 patients were used for the validation of the OPSCC miRNA prognostic model and 39 patients were used for the validation of the OPSCC mRNA model.

### Quantitative reverse‐transcription PCR for miRNA model validation

Quantitative reverse‐transcription polymerase chain reaction (qRT‐PCR) was used to profile the miRNAs identified as significant in OPSCC. The details of this experimental procedure have been described previously 17. Briefly, the RT reaction was performed with the High Capacity cDNA Reverse Transcription Kit (Life Technologies, Foster City, CA, USA). Each RT reaction included 100 ng of tumor RNA and a pool of RT primers for selected miRNAs and control RNAs. Quantitative PCR was performed with Power SYBR Green PCR Master Mix (Life Technologies) and specific PCR primers for selected miRNAs or control RNAs. miRNA raw profiling data for individual samples were normalized with four small RNA controls (SNORD48, SNORD47, RNA5‐8S5, and RNU6‐1). Specifically, the expression levels of the four small RNAs were averaged and used as the reference to control for sample variations during miRNA profiling analysis.

The expression of p16 protein was determined by immunohistochemistry as previously described 18. The expression profiles of E6 and E7 transcripts from six oncogenic HPV types were determined by qRT‐PCR, including types 16, 18, 33, 39, 56, and 59. The details of the HPV assays and the experimental protocol have been described previously 18. In brief, primer sequences for the assays were selected from the E6 and E7 coding regions of the high‐risk HPV genomes. The expression profiles of GAPDH and *β*‐actin were used as reference controls for data normalization.

## Results

### Validation of an existing miRNA prognostic signature

To verify whether the miRNA data obtained from The Cancer Genome Atlas (TCGA) could be used in further biomarker identification, we evaluated our previously published prognostic model for OPSCC 8 with TCGA data. Briefly, this model identified miR‐24‐3p, miR‐31‐5p, and miR‐193b‐3p as negatively associated with survival, and miR‐26b‐5p, miR‐142‐3p, and miR‐146a‐5p as positively associated with survival. The expression levels of these six miRNAs were then combined to create a single prognostic model as described previously 8:

$$\begin{array}{cc} & S\;=\;2.62E_{\mathrm{miR}-24-3p}+3.16E_{\mathrm{miR}-31-5p}+2.45E_{\mathrm{miR}-193b-3p}\\ & \;-2.69E_{\mathrm{miR}-26b-5p}-3.34E_{\mathrm{miR}-142-3p}-2.81E_{\mathrm{miR}-146a-5p} \end{array}$$

**Figure 1:**
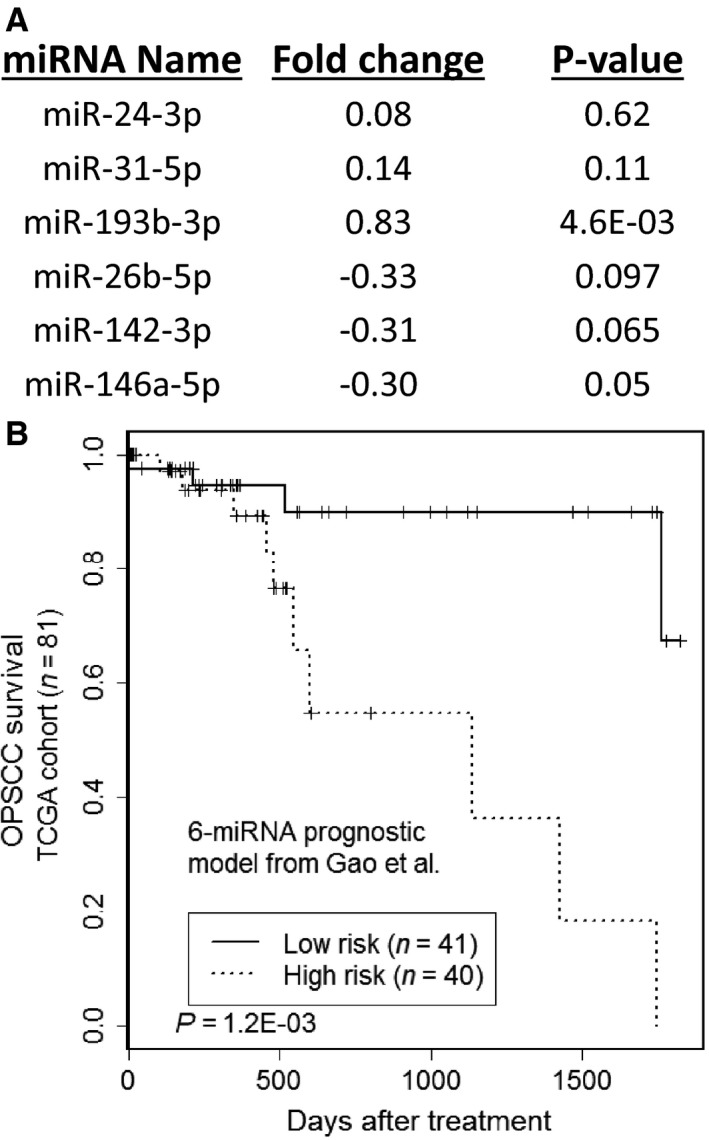
Validation of an existing miRNA signature with TCGA data. (A) The six miRNAs from our previously published prognostic model for OPSCC were examined for association with the overall survival of TCGA patients. Fold change values were log_2_ transformed and represent the average expression difference of the miRNAs in the deceased patient group compared to the living patient group. Statistical significance was determined with the log‐rank test in Cox regression analysis. (B) Kaplan–Meier survival analysis to evaluate the prognostic performance of the six‐miRNA signature for predicting overall survival in OPSCC.

We examined each miRNA individually with TCGA data and found that two miRNAs were significantly associated with survival, two miRNAs maintained borderline significance (*P* < 0.1), and two miRNAs was not found to be significant (*P* > 0.1) (Fig. 1A). It should be noted, however, that the directions of expression changes in relation to survival outcome were maintained for all six miRNAs (i.e., positive correlations for miR‐26b‐5p, miR‐142‐3p, miR‐146a‐5p, and negative correlations for miR‐31‐5p, miR‐193b‐3p, miR‐26b‐5p) (Fig. 1A). When we analyzed this prognostic model as whole, it was able to significantly differentiate between high‐ and low‐risk OPSCC patients from TCGA (Fig. 1B). This demonstrated that this previously published model was robust and could be applied to patient miRNA profiles obtained from other institutions, while also indicating that the data from TCGA was a valuable resource for further biomarker identification and analysis.

### Unique TCGA miRNA expression profiles correlated with overall survival in OPSCC

miRNA expression analysis was performed for the 81 OPSCC patients obtained from TCGA. The characteristics of these patients are summarized in Table 1. The miRNAs were examined individually using Cox univariate proportional hazards analysis to determine which miRNAs were correlated with overall survival. This analysis provides a log‐rank *P*‐value, which indicates the significance of the miRNA in relation to survival, as well as a Wald coefficient, which indicates the weight associated with the expression level of the miRNA.

**Table 1:** Characteristics of the HNSCC patients included in TCGA

Characteristics	OPSCC (*n* = 81)	OSCC (*n* = 311)	LSCC (*n* = 115)
Age at diagnosis (mean ± SD, years)	55.9 ± 9.3	61.9 ± 13.2	61.9 ± 9.1
Sex			
Male	69 (85.2%)	206 (66.2%)	95 (82.6%)
Female	12 (14.8%)	105 (33.8%)	20 (17.4%)
Race			
White	75 (92.6%)	268 (86.2%)	91 (79.1%)
Other	6 (7.4%)	43 (13.8%)	24 (20.9%)
Smoking^a^			
Unreported	1 (1.2%)	10 (3.2%)	4 (3.5%)
Nonsmoker	25 (30.8%)	88 (28.3%)	6 (5.2%)
Long‐term former smoker	8 (9.9%)	51 (16.4%)	11 (9.6%)
Other former smoker	25 (30.9%)	68 (21.9%)	36 (31.3%)
Current smoker	22 (27.2%)	94 (30.2%)	58 (50.4%)
T classification			
T1	13 (16.0%)	29 (9.3%)	7 (6.1%)
T2	36 (44.4%)	102 (32.8%)	20 (17.4%)
T3	20 (24.7%)	64 (20.6%)	33 (28.7%)
T4	12 (14.8%)	116 (37.3%)	55 (47.8%)
N Classification			
NX	0 (0.0%)	4 (1.3%)	2 (1.7%)
N0	21 (25.9%)	142 (45.7%)	52 (45.2%)
N1	52 (64.2%)	52 (16.7%)	12 (10.4%)
N2	3 (3.7%)	110 (35.4%)	46 (40.0%)
N3	5 (6.2%)	3 (1.0%)	3 (2.6%)
Stage			
I	5 (6.2%)	19 (6.1%)	2 (1.7%)
II	11 (13.6%)	62 (19.9%)	15 (13.0%)
III	12 (14.8%)	57 (18.3%)	18 (15.7%)
IV	53 (65.4%)	173 (55.6%)	80 (69.6%)
Deceased	14 (17.2%)	109 (35.0%)	33 (28.7%)

OPSCC, oropharyngeal squamous cell carcinoma; OSCC, oral cavity squamous cell carcinoma; LSCC, laryngeal squamous cell carcinoma; SD, standard deviation.

a Smoking was defined as no history of smoking, a former smoker of ≥15 years, other former smoker of <15 years, or a current smoker.

We then implemented recursive feature elimination (RFE) technique to determine the relative prognostic performance of individual miRNAs. In this process, a regression model was generated using the given miRNA features and outcomes (i.e., miRNA expression and overall survival, respectively), and the least impactful feature was eliminated. The process was then repeated until the final iteration identified the most significant feature associated with the classifier. This was performed on a subset of top‐ranking 189 miRNAs in OPSCC ordered by log‐rank *P*‐value while maintaining a Wald coefficient greater than or equal to one, and an average expression across all samples greater than 1.414 (i.e., a log_2_ Expression of 0.5). In this way, we were able to initially identify a set of promising miRNA candidates for further model development.

**Figure 2:**
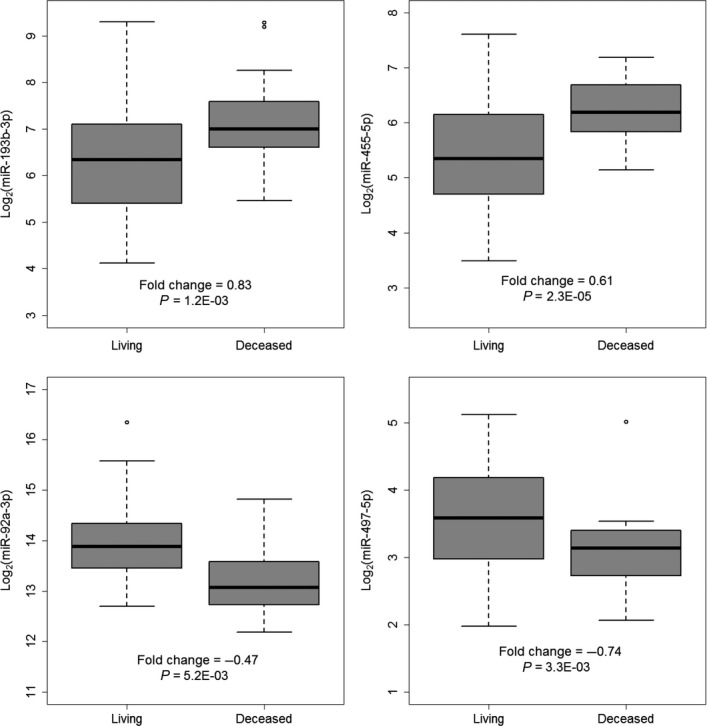
Four significant miRNAs associated with overall survival of TCGA OPSCC patients. Fold change values were log_2_ transformed and represent the average expression difference of the miRNAs in the deceased patient group compared to the living patient group. Significance was determined with the log‐rank test in Cox regression analysis.

In examining the 50 most significant miRNAs in accordance with the RFE, miR‐193b‐3p, miR‐455‐5p, miR‐92a‐3p, and miR‐497‐5p were identified as maintaining a high RFE ranking after 10‐fold cross‐validation, as well as being statistically significant in the univariate Cox proportional hazards analysis (Fig. 2). All four of these miRNAs have been reported as dysregulated in other cancer types, including colorectal cancer and pancreatic cancer 19, 20, 21, 22. In particular, the validity of miR‐193b‐3p as a prognostic marker in OPSCC was previously reported and incorporated in our previous model for outcome prediction 8.

### A combined miRNA prognostic signature predicts overall survival in OPSCC

We further hypothesized that a combination of prognostic miRNAs within OPSCC could be effectively used to predict overall survival. The miRNAs chosen in the aforementioned analysis were used to build the following prognostic model:

$$\begin{array}{cc} & S_{\mathrm{OPSCC}}=11.31E_{\mathrm{miR}-193b-3p}+13.53E_{\mathrm{miR}-455-5p}\\ & \;-7.25E_{\mathrm{miR}-92a-3p}-7.3E_{\mathrm{miR}-497-5p}, \end{array}$$

where *S* indicates the risk score for each patient and *E* represents the normalized expression level of the identified miRNA from the primary tumor. The coefficients in this equation are the Wald scores from the Cox regression analysis and are representative of the relative importance of the miRNA toward survival status.

**Figure 3:**
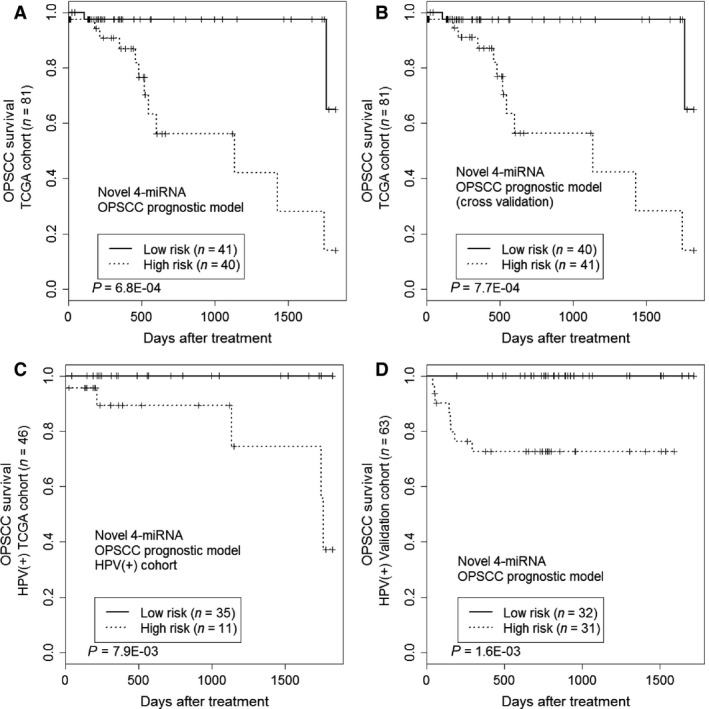
Kaplan–Meier survival analysis to evaluate the novel OPSCC 4‐miRNA prognostic signature. Patients were stratified into the low‐risk group or high‐risk group based on risk score. (A) The signature was evaluated for overall survival in the training set from TCGA. Significance was determined using the log‐rank test. (B) Leave‐one‐out cross‐validation to evaluate the miRNA modeling strategy. The cross‐validated results from all rounds were combined for prognostic evaluation of overall survival. (C) Independence of the miRNA signature in HPV(+) patients. (D) Survival analysis to evaluate the miRNA signature for overall survival in the validation cohort.

In this prediction model, higher scores indicate higher risk and predict a poor survival outcome for the patient. The patients were stratified internally by median risk score to produce two cohorts of similar size, so as to determine the validity of the prognostic model. By this method, 40 OPSCC patients were predicted to be high‐risk (with > median score) and 41 patients were predicted to be low‐risk (i.e., with ≤ median score); significantly different risks of death were observed based on this classification (*P* = 6.8E‐04) (Fig. 3A).

One primary concern for prognostic model development is the risk of overtraining. To address this issue, we performed leave‐one‐out cross‐validation. For this cross‐validation, within each iteration, we removed one sample from the training set and trained a model with the miRNA profiles from the remaining samples. The removed sample was then used for independent model testing. The process was repeated until all the samples had been used independently for model testing. For each validation round, the Wald coefficient for the candidate miRNAs were calculated based on the training set and used to generate a slightly different model for testing. Cross‐validation still yielded a significant separation of high‐ and low‐risk patients (Fig. 3B), indicating that the model is robust within the training data.

### The miRNA prognostic signature was independent of clinical features

We assessed whether the miRNA signature maintained its prognostic value within the context of commonly used clinical parameters, including age at diagnosis, gender, race, smoking history, initial tumor staging, and treatment type. This analysis was conducted through multivariate Cox hazards analysis. This miRNA signature was found to maintain statistical significance, with a hazards ratio of 11.85 and *P*‐value of 3.9E‐03 (Table S1).

### The OPSCC miRNA signature maintained its prognostic value independent of HPV status

Previous work has shown that HPV positivity is a favorable prognostic marker in OPSCC, and thus we extended our miRNA signature to explore whether the prognostic significance was maintained independent of HPV status. OPSCC patients were identified as HPV‐positive if sequencing reads from the RNA‐seq data that did not align to the human genome aligned to any of the 143 types of HPV. Of the 72 OPSCC patients with RNA‐seq data, 46 were identified as having reads aligned to one of three types of HPV. Specifically, 39 patients were positive for HPV16, four for HPV33, and three for HPV35, leaving 26 patients as HPV‐negative.

Of the 46 patients who were identified as HPV‐positive, 35 were identified as low‐risk and 11 as high‐risk by the miRNA prognostic signature. Kaplan–Meier survival analysis indicated that the high‐risk group had poor survival as compared to the low‐risk group (*P* = 7.9E‐03) (Fig. 3C). The model was not statistically significant when applied to HPV‐negative patients (data not shown); however, it should be noted that the HPV‐negative set was a much smaller cohort (*n* = 26), which significantly reduced the power of the model.

### Validation of the OPSCC miRNA signature with an independent cohort

To confirm the validity of the 4‐miRNA model for OPSCC prognosis, we applied our miRNA signature to an independent cohort of 66 OPSCC patients treated at the Washington University School of Medicine in St. Louis. The clinical characteristics of these patients are outlined in Table S2. We hypothesized that the miRNA signature provides independent prognostic value from HPV biomarker. Since HPV positivity is a favorable prognostic marker for OPSCC, we were interested to know whether the new miRNA signature maintains its prognostic value by further risk‐stratifying HPV‐positive patients.

All 66 patients were preselected to be p16‐positive by immunohistochemistry, as p16 is a robust surrogate biomarker for HPV expression 18. HPV expression in these tumors was further validated by quantitative reverse‐transcription PCR (qRT‐PCR, see Materials and Methods for details). Of the 66 tumors, 61 were HPV16‐positive and two were HPV18‐positive. HPV transcripts were not detected in the remaining three samples.

Furthermore, qRT‐PCR was conducted on the tumor samples for the four miRNAs included in the signature. miRNA expression readings were normalized using four internal small RNA controls (see Materials and Methods for details). The risk score was then calculated for each of these patients based on the miRNA signature. The patients were then stratified into high‐risk and low‐risk groups by the median risk score. Kaplan–Meier survival analysis indicated that the miRNA model was significantly predictive of survival outcome for the 63 HPV‐positive cases (*P* = 1.6E‐03, Fig. 3D). The miRNA signature had a similar prognostic performance when applied to all 66 p16‐positive cases (*P* = 2.8E‐03).

We also analyzed the signature prediction scores with a receiver operating characteristic curve, which evaluated both the true positive rate (sensitivity) and the false positive rate (specificity). In the training and validation sets, the areas under the curve were 0.84 and 0.84, respectively, indicating robust performance of the model for both sensitivity and specificity when applied to independent cohorts (Figure S1).

### Unique miRNA expression profiles correlated with distinct subtypes of head and neck cancer

We extended our miRNA expression profiling analysis to the 311 OSCC and 115 LSCC patients obtained from TCGA. The characteristics of these patients are summarized in Table 1. For each additional subtype of HNSCC, we conducted similar analyses as described for OPSCC and identified four miRNAs in each subset that were predictive of overall survival (Table S5). These miRNAs were then combined to generate the following prognostic models:

$$\begin{array}{cc} & S_{\mathrm{OSCC}}=10.73E_{\mathrm{miR}-337-3p}+7.82E_{\mathrm{miR}-369-5p}\\ & \;+6.21E_{\mathrm{miR}-218-5p}+7.01E_{\mathrm{miR}-127-5p}, \end{array}$$

$$\begin{array}{cc} & S_{\mathrm{LSCC}}=-10.70E_{\mathrm{let}-7a-3p}-6.96E_{\mathrm{miR}-145-5p}\\ & \;+4.59E_{\mathrm{miR}-129-5p}-6.43E_{\mathrm{miR}-26b-5p} \end{array}$$

**Figure 4:**
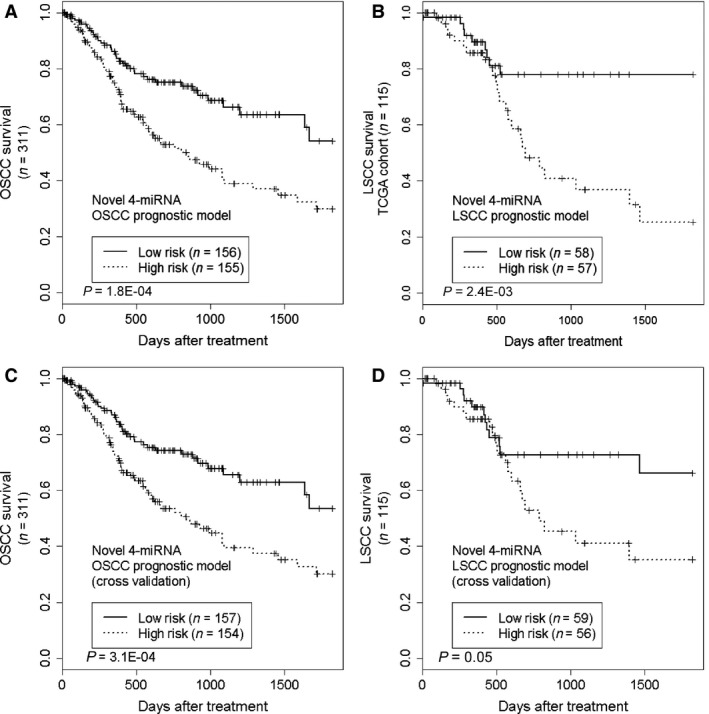
Kaplan–Meier survival analysis to evaluate the OSCC and LSCC miRNA prognostic models. (A, B) The models were evaluated in the respective training sets. (C, D) Leave‐one‐out cross‐validation results were combined for prognostic evaluation.

As described earlier, the median score was used within each subset to separate patients into high‐ and low‐risk groups, which were also found to have significantly different risks of death (*P* = 1.8E‐04 in OSCC and *P* = 2.4E‐03 for LSCC) (Fig. 4A and B). We also conducted leave‐one‐out cross‐validation analysis for these two signatures and found a significantly different risk of survival in the miRNA‐stratified groups of OSCC and a borderline significance for LSCC (Fig. 4C and D). Despite borderline significance of the LSCC model in cross‐validation analysis, the LSCC prognostic miRNA model may still be useful for prediction of patient survival. In particular, these models maintained statistical significance independent of clinical features when analyzed with multivariate Cox analysis, with the OSCC model having a hazards ratio of 1.88 and a *P*‐value of 1.8E‐03, and the LSCC model having a hazards ratio of 2.84 and a *P*‐value of 1.3E‐02 (Table S1).

It is noteworthy that each miRNA signature carried prognostic significance when applied to the HNSCC subtype where it was derived. On the other hand, when applied to other subtypes of HNSCC, none of the signatures were able to effectively distinguish high‐risk and low‐risk patients (Figure S2). We also observed this phenomenon when we applied the previously developed 6‐miRNA prognostic model to OSCC and LSCC (Figure S3). In conjunction with previous studies indicating significant genetic heterogeneity between subtypes of HNSCC 10, our results indicate that the miRNome is just as unique for each HNSCC subtype.

## Discussion

Identification of novel prognostic biomarkers typically requires a large dataset, which provides sufficient statistical power for discovery research. To this end, we took advantage of the high‐throughput data from TCGA for biomarker analysis. The TCGA consortium has published many studies identifying the mutations and dysregulations associated with tumors in comparison to matched normal tissue samples. There are also a number of studies that used TCGA data for independent validation of existing biomarkers 23, 24, 25. Additionally, many studies exploring miRNA biomarkers in head and neck cancer, such as the miR‐34 family and miR‐200a, have indicated their roles in oncogenesis 26. However, few studies have utilized TCGA data in systematically identifying biomarkers associated with patient outcome.

In this study, we have presented a new strategy to identify prognostic miRNA biomarkers by analyzing TCGA data directly, followed by experimental validation using an independent cohort. As the first step, we utilized TCGA data as the primary source to identify biomarkers and develop prognostic models for OPSCC. Within OPSCC, infection by HPV has already been indicated as a favorable prognostic factor 6. Our model was able to further improve the prognostic value of HPV positivity by identifying a high‐risk cohort among HPV(+) patients. Next, we were able to validate the robustness of this signature using an independent cohort that consisted only of HPV(+) OPSCC patients. This confirmed that the miRNA signature was able to further distinguish high‐ and low‐risk patients within HPV(+) OPSCC patients.

Among the subtypes of HNSCC, OPSCC has unique characteristics as HPV infection is associated with most OPSCC cases. Although the total number of HNSCC cases has decreased steadily on a yearly basis, the number of reported OPSCC cases has increased significantly as a result of rapid rise in HPV(+) OPSCC cases 27, 28. Our clinical goal of building a powerful prognostic model is to reliably stratify OPSCC patients for treatment failures after standard therapy. The availability of such a reliable prognostic model is critical for providing individualized cancer therapy, including both deintensifying treatment for low‐risk patients as well as intensification for high‐risk patients. In particular, there is currently significant clinical interest in identifying a subset of OPSCC patients who have low‐risk of treatment failures, in order to deintensify their overall treatment. As present, multi‐institutional de‐escalation clinical trials are underway for HPV(+) OPSCC patients 29, 30. However, there is still a significant portion of HPV(+) OPSCC cases that have poor outcome. For these cases, de‐escalation treatment should not be applied and instead the treatment should be intensified. Thus, there is a critical need to develop robust prognostic models to further stratify HPV(+) OPSCC patients for enrollment in de‐escalation trials. To this end, our proposed miRNA‐based prognostic model will fill in a critical need by selecting HPV(+) OPSCC patients who will most likely benefit from de‐escalation treatment. Further work would be required to bring this signature fully to the clinical setting, such as the inclusion of robust reference genes to standardize the signature scores, which allows clinicians to determine the appropriate treatment modality based a predefined threshold score.

Besides OPSCC, we have also shown that our strategy on TCGA‐based biomarker discovery can be extended to the study of other subtypes of HNSCC. In this way, we have demonstrated that TCGA represents a rich resource for cancer prognostic studies. We expect that prognostic tools developed using TCGA data, with proper validation, will significantly expand our ability to more precisely manage cancer patients by applying individualized treatment plans.

## Conflicts of Interest

None.

## Supporting information


**Data S1.** Combined supplementary methods and results.
