# Supplementary material for: Prognostic microRNA signatures derived from The Cancer Genome Atlas for head and neck squamous cell carcinomas
Source: Cancer Med. 2016 Apr 25;5(7):1619–28. doi: 10.1002/cam4.718 (PMC4944889; doi:10.1002/cam4.718)

Supplementary Table 1. Multivariate Cox regression analysis to evaluate independence of the prognostic miRNA signatures from clinical parameters.

**Parameter**	**OPSCC**		**OSCC**		**LSCC**	
	HR	P-value [a]	HR	P-value [a]	HR	P-value [a]
miRNA signature	11.847	0.0039	1.88	1.8E-03	2.843	1.3E-02
Age	1.056	0.054	1.017	6.1E-02	0.978	0.32
Sex	0.741	0.68	1.129	0.6	0.455	5.6E-02
Stage (I/II/III vs IV)	2.936	0.11	2.155	6.3E-04	1.013	0.91
Tobacco	1.386	0.31	1.052	0.54	1.17	0.40
Treatment (chemotherapy vs radiotherapy vs combined)	0.637	0.034	0.906	0.96	0.866	0.17
Race (White vs. other)	7.906	0.11	0.919	0.95	2.369	2.6E-02

[a] P-values were calculated using the Wald test.

Supplementary Table 2. Characteristics of the OPSCC patients at Washington University.

	**OPSCC miRNA validation cohort (n=66)**	**OPSCC mRNA gene validation cohort (n=39)**
**Age at diagnosis (mean ± SD, y)**	58.5 ± 10.2	55.8 ± 10.3
**Sex**		
Male	54 (81.8%)	36 (92.3%)
Female	12 (18.2%)	3 (7.7%)
**Race**		
White	65 (98.5%)	36 (92.3%)
Other	1 (1.5%)	3 (7.7%)
**Smoking**		
Unreported	1 (1.5%)	4 (10.3%)
Non-smoker	22 (33.3%)	12 (30.8%)
Former smoker	27 (40.9%)	20 (51.3%)
Current smoker	16 (24.2%)	3 (7.7%)
**T Classification**		
Tx	6 (9.1%)	6 (15.4%)
T1	28 (42.4%)	14 (34.9%)
T2	15 (22.7%)	9 (23.1%)
T3	7 (10.6%)	4 (10.3%)
T4	10 (15.2%)	6 (15.4%)
**N Classification**		
NX	0 (0.0%)	6 (15.4%)
N0	12 (18.2%)	2 (5.1%)
N1	13(19.7%)	4 (10.2%)
N2	37 (56.1%)	24 (61.5%)
N3	4 (6.1%)	3 (7.7%)
**Stage**		
Unreported	0 (0.0%)	6 (15.4%)
I	4 (6.1%)	1 (2.6%)
II	5 (7.6%)	0 (0.0%)
III	15 (22.7%)	5 (12.8%)
IV	42 (63.6%)	27 (69.2%)
**Deceased**	10 (15.2%)	14 (35.9%)

Supplementary Table 3. Genes identified as significantly dysregulated between living and deceased patients by combined SVM-RFE and Cox regression analysis.

Gene ID	Gene abbreviation	p-value [a]	Fold change [b]	Wald coefficient [c]
490	ATP2B1	4.0E-03	-0.611	-6.15
926	CD8B	4.9E-04	-1.513	-5.00
1386	ATF2	7.4E-04	-0.405	-9.90
3963	LGALS7	5.5E-03	0.480	6.34
4103	MAGEA4	9.2E-03	2.685	8.40
4322	MMP13	9.3E-04	2.862	12.83
5412	UBL3	3.5E-05	-0.437	-10.67
8829	NRP1	3.5E-03	-0.223	8.46
10362	HMG20B	3.1E-03	-0.416	-6.85
10868	USP20	3.6E-05	-0.475	-10.43
11044	PAPD7	2.4E-04	-0.527	-11.21
22887	FOXJ3	4.2E-05	-0.367	-14.87
23530	NNT	1.3E-04	-0.661	-11.88
51104	ABHD17B	4.3E-06	-0.631	-15.28
51350	KRT76	5.6E-03	0.961	11.51
55450	CAMK2N1	1.2E-03	1.171	12.63
56267	CCBL2	1.2E-04	-0.770	-11.73
64062	RBM26	2.6E-04	-0.452	-9.79
64766	S100PBP	1.5E-05	-0.393	-13.69
64781	CERK	6.0E-04	-0.462	-11.42
101928783	LOC10192783	6.0E-03	0.743	7.04

[a] The p-values were calculated using the logrank test in univariate Cox proportional hazards analysis.
[b] Fold change values were $\log_2$ transformed, representing the average expression difference of the miRNAs in the 2 patient groups (deceased vs alive).
[c] The Wald coefficient was obtained from the Wald test in univariate Cox proportional hazards analysis.

Supplementary Table 4. Correlation of the identified OPSCC genes with prognostic miRNA biomarkers.

		**miR-92a**	**miR193b**	**miR-455**	**miR-497**
4103	MAGEA4	-0.32	0.33	0.19	-0.09
56267	CCBL2	-0.08	-0.24	-0.21	0.07
51104	ABHD17B	-0.16	-0.19	-0.15	-0.12
64766	S100PBP	0.02	-0.26	-0.13	0.09
10868	USP20	-0.01	-0.22	-0.17	0.13
22887	FOXJ3	-0.03	-0.11	-0.10	-0.05
64062	RBM26	0.22	-0.22	-0.09	0.04
490	ATP2B1	-0.06	-0.01	-0.18	-0.09
8829	NRP1	-0.44	0.08	0.04	-0.12
3963	LGALS7	-0.33	0.19	0.28	-0.17
101928783	LOC10192783	-0.18	0.13	0.11	-0.24
4322	MMP13	-0.45	0.17	0.21	-0.14
1386	ATF2	-0.04	0.05	-0.04	-0.15
23530	NNT	-0.03	-0.22	-0.10	-0.05
10362	HMG20B	-0.21	-0.07	0.00	0.01
5412	UBL3	-0.16	-0.19	-0.23	-0.05
55450	CAMK2N1	-0.40	0.34	0.31	-0.15
51350	KRT76	-0.37	0.25	0.27	-0.22
11044	PAPD7	0.02	-0.08	0.00	-0.04
926	CD8B	0.07	0.01	0.06	0.14
64781	CERK	-0.11	-0.18	-0.15	-0.01

The numbers represent correlation coefficients between individual miRNAs and mRNA genes. The underlined scores indicate that the gene is identified as a target of the associated miRNA based on miRDB prediction.

Supplementary Table 5. Significantly dysregulated miRNAs associated with overall survival and used to develop prognostic models for OSCC and LSCC, respectively.

	miRNA name	Fold change [a]	p-value [b]
**OSCC**	hsa-miR-337-3p	0.220	8.6E-04
	hsa-miR-369-5p	0.428	5.5E-03
	hsa-miR-218-5p	0.197	1.4E-02
	hsa-miR-127-5p	0.381	7.0E-03
**LSCC**	hsa-let-7a-3p	-0.710	5.2E-04
	hsa-miR-145-5p	-0.440	6.2E-03
	hsa-miR-129-5p	1.349	3.8E-02
	hsa-miR-26b-5p	-0.333	8.4E-03

Supplementary Methods

RNA sequencing for mRNA gene model validation

RNA sequencing (RNA-seq) was utilized to profile the expression of the identified mRNA biomarkers from TCGA analysis. Details of the experimental protocol have been described previously [1]. Briefly, total RNA was used to construct cDNA libraries which were then provided to the Genome Technology Access Center at Washington University School of Medicine for sequencing with Illumina HiSeq 2500. The sequence reads were preprocessed to remove low-quality reads before being aligned to the human transcriptome and virome, as described previously.

Supplementary Results

Identification of an mRNA-based prognostic signature for overall survival in OPSCC

Of the 82 identified OPSCC patients, 72 had raw RNA-seq data available. This dataset was analyzed to determine which genes were significantly over- or under-expressed when associated with overall survival. In this way, we identified 21 genes that showed significant correlation to survival based on univariate Cox analysis (Supplementary Table 3). These genes were also identified as having high relative independent prognostic values through RFE, as described earlier.

To confirm that these genes were not associated with the miRNA signature, we performed correlation analysis against the four miRNAs included in the prognostic signature. These genes showed little negative correlation to the four prognostic miRNAs, suggesting that the genes were not directly regulated by the miRNAs (Supplementary Table 4). We also confirmed the lack of regulatory relationship between the miRNAs and mRNAs by target prediction analysis through miRDB [2](data not shown).

Similar to the miRNA signature, an mRNA prognostic signature for OPSCC was developed using these 21 genes. The coefficients of the model were determined using the Wald scores from the univariate Cox analysis. As described earlier, the patients were stratified into high-risk and low-risk groups by the median score, and the two cohorts showed significantly different likelihood of survival (p = 1.1E-06) (Supplementary Figure 4A). This indicates that within the training set obtained from TCGA, this mRNA signature was predictive of survival outcome.

Evaluation of the mRNA-based signature with an independent dataset

The mRNA signature was evaluated in an independent HPV-positive OPSCC cohort obtained from Washington University School of Medicine. The clinical characteristics of these patients are outlined in Supplementary Table 2. Within this 39 patient cohort, 14 were deceased by the end of five years after treatment. The mRNA signature was evaluated with this cohort through RNA-seq expression analysis. The patients were stratified by median risk score, resulting in 19 patients classified into the high-risk population and 20 into the low-risk population. The mRNA signature was not validated with this independent cohort, as the patients stratified by the signature had very similar outcomes in overall survival (p=0.77) (Supplementary Figure 4B).

Supplementary Discussion

Besides the miRNA-seq profiling data, we have also analyzed the RNA-seq data from TCGA to develop an mRNA-based gene signature for OPSCC prognosis. However, this signature was not validated with independent data. This possibly reflected more expression variations for mRNAs in comparison to miRNAs, or may be the result of a smaller sample size, which led to decreased statistical power. Although the mRNA signature was not validated, the mRNA expression data obtained for OPSCC still provided valuable information for functional analysis. Upon conducting gene set enrichment analysis [3], we found that within the deceased patient

group, the genes associated with epithelial-mesenchymal transition were significantly upregulated (data not shown). This particular cell function has been characterized as crucial in tumor progression [reviewed in [4, 5]] and may provide insights into which additional genomic features are involved in the progression of OPSCC. Improved understanding of these mechanisms may lead to further advances in patient prognosis and treatment.

Supplementary References

1. Jiang Z, Liu W, Wang Y, Gao Z, Gao G, Wang X. Rational design of microRNA-siRNA chimeras for multifunctional target suppression. *RNA.* 2013;19:1745-1754.
2. Wong N, Wang X. miRDB: an online resource for microRNA target prediction and functional annotations. *Nucleic Acids Res.* 2015;43:D146-D152.
3. Subramanian A, Tamayo P, Mootha VK, et al. Gene set enrichment analysis: A knowledge-based approach for interpreting genome-wide expression profiles. *Proc Natl Acad Sci U S A.* 2005;102:15545-15550.
4. Thiery JP. Epithelial-mesenchymal transitions in tumour progression. *Nat Rev Cancer.* 2002;2:442-454.
5. Kalluri R, Weinberg RA. The basics of epithelial-mesenchymal transition. *J Clin Invest.* 2009;119:1420-1428.

Supplementary Figure Legends

Supplementary Figure 1. Receiver operating characteristic (ROC) curves for the training cohort from TCGA and the validation cohort consisting of HPV-positive cases from Washington University.

Supplementary Figure 2. Kaplan-Meier survival analysis to evaluate the miRNA prognostic signatures in other subtypes of HNSCC. Survival analysis of the OPSCC miRNA signature in OSCC **(A)** and LSCC **(B)**; survival analysis of the OSCC miRNA signature in OPSCC **(C)** and LSCC **(D)**; survival analysis of the LSCC miRNA signature in OSCC **(E)** and LSCC **(F)**.

Supplementary Figure 3. Kaplan-Meier survival analysis to evaluate an existing OPSCC miRNA signature in OSCC **(A)** and LSCC **(B)**.

Supplementary Figure 4. Kaplan-Meier survival analysis to evaluate the mRNA prognostic signature for overall survival in the training cohort **(A)** and the validation cohort **(B)**.

Supplementary Figure 1

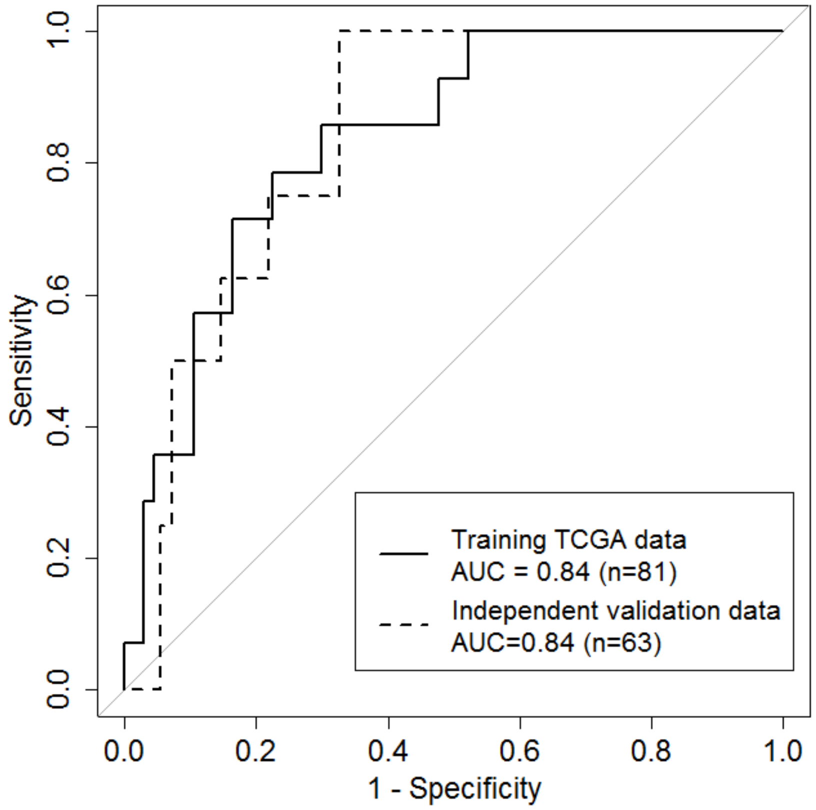

Supplementary Figure 2

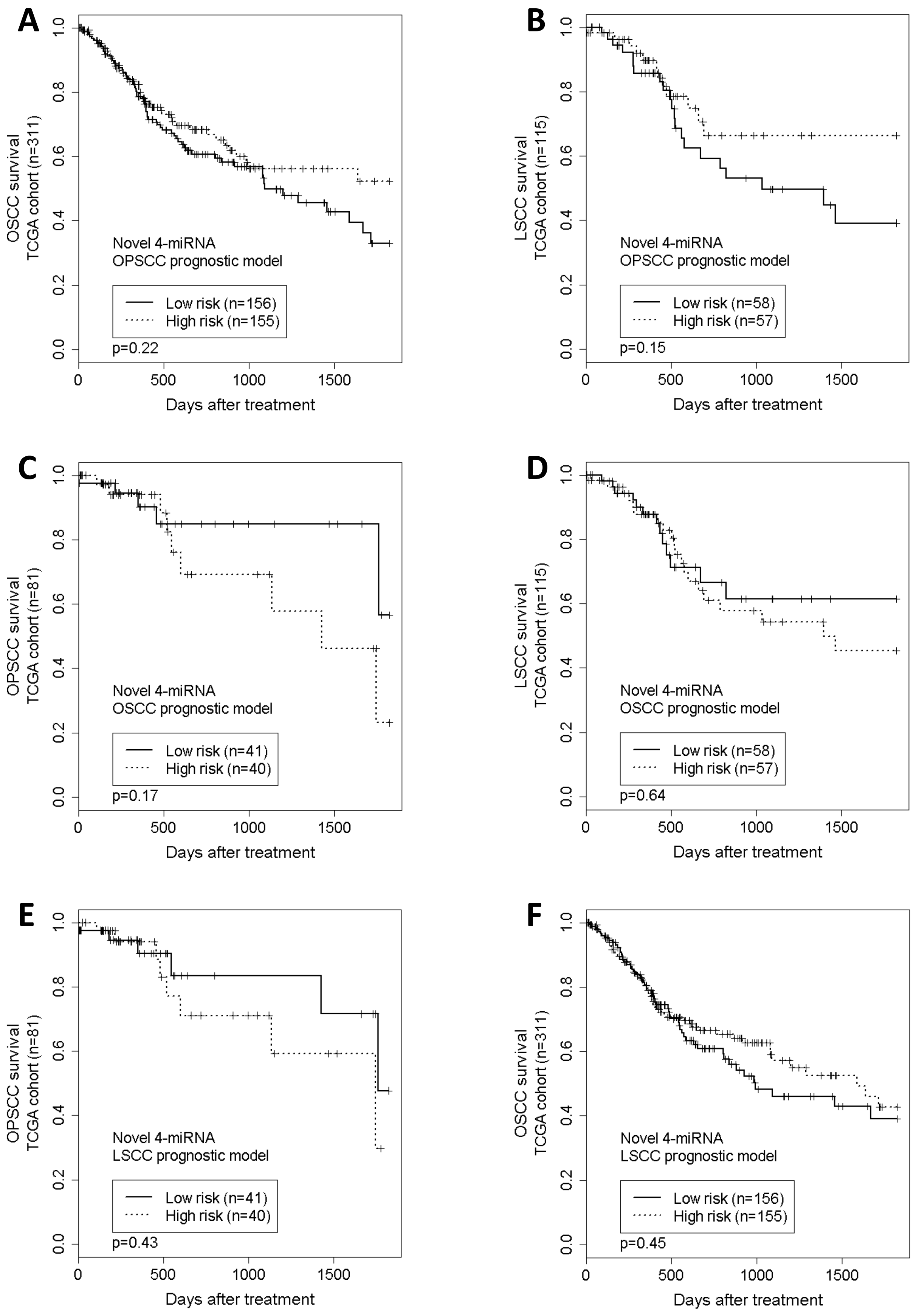

Supplementary Figure 3

A

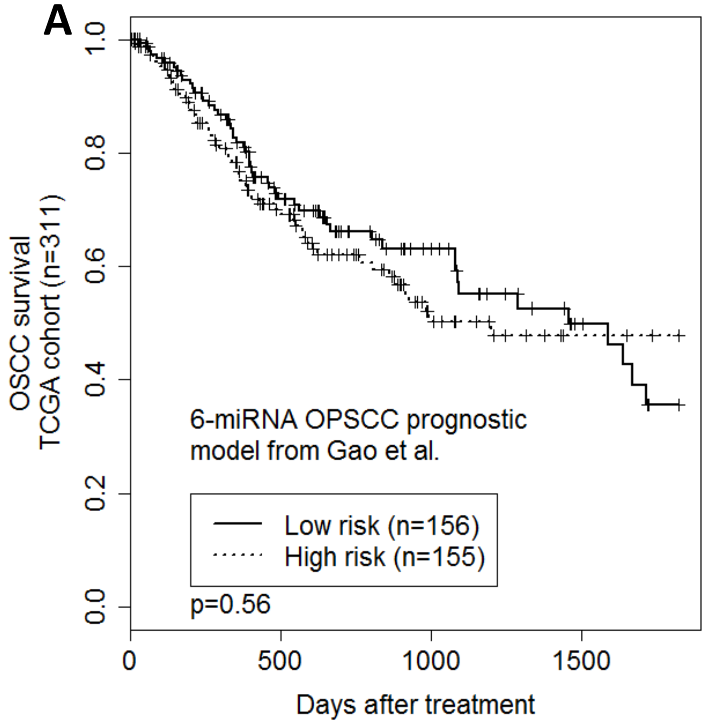

B

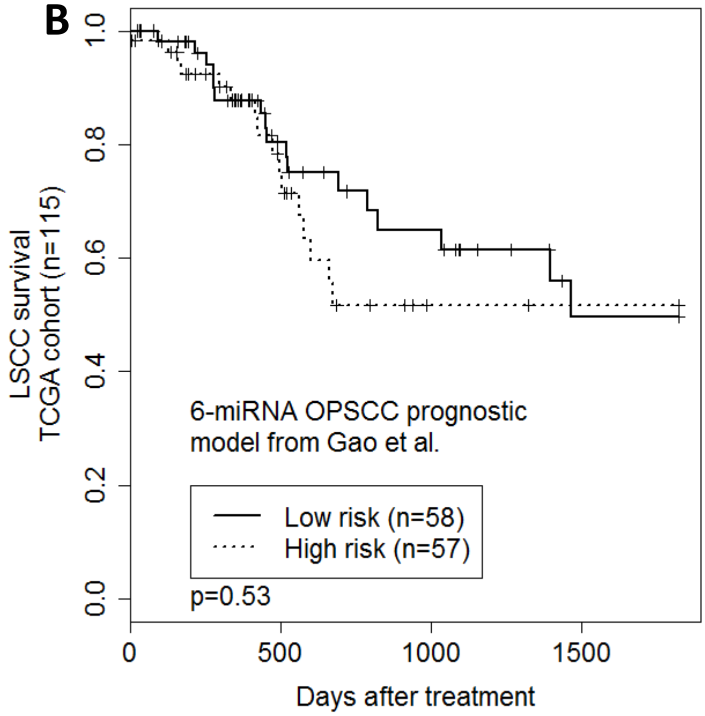

Supplementary Figure 4

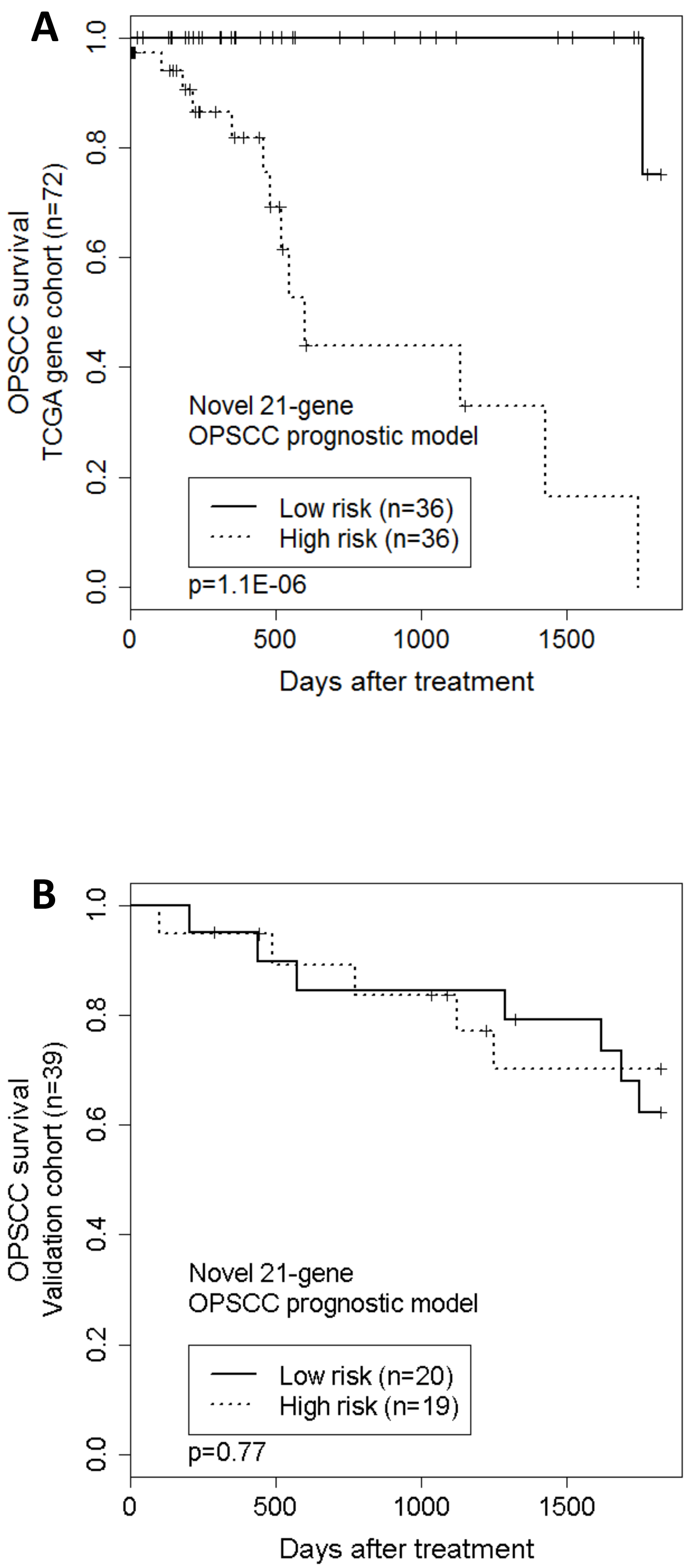

Supplement: Supplementary file 1 — Data S1. Combined supplementary methods and results. [file CAM4-5-1619-s001.pdf]
